# Cavernous mediastinal hemangioma presenting with persistent cough: a rare case report and review of literature

**DOI:** 10.1186/s13019-023-02130-7

**Published:** 2023-01-05

**Authors:** Parviz Mardani, Hooman Kamran, Bita Geramizadeh, Mohammad Hassan Darabi, Masoud Najafi, Armin Amirian, Reza Shahriarirad

**Affiliations:** 1 Thoracic and Vascular Surgery Research Center, Shiraz University of Medical Science, Shiraz, Iran; 2 Students Research Committee, School of Medicine, Shiraz University of Medical Sciences, Shiraz, Iran; 3 Shiraz Transplant Research Center (STRC), Shiraz University of Medical Sciences, Shiraz, Iran; 4 Department of Pathology, Shiraz University of Medical Sciences, Shiraz, Iran; 5 Maternal-Fetal Medicine Research Center, Shiraz University of Medical Sciences, Shiraz, Iran; 6 School of Medicine, Shiraz University of Medical Sciences, Shiraz, Iran

**Keywords:** Case report, Cavernous hemangiomas, Hemangioma, Mediastinal neoplasms, Mediastinum

## Abstract

**Background:**

Cavernous hemangioma is a rare benign tumor which can sometimes mimic the clinical presentation and radiological findings of malignant tumors. Here we present a rare presentation of cavernous hemangioma in the mediastinum (CHM), along with a literature review among the main databases.

**Case presentation:**

We present a 48-year-old male who had suffered from persistent cough as the sole symptom of an anterior CHM. Computed tomography scan demonstrated a 12.5 × 10.8 cm mass in the anterior mediastinum. The mass was surgically resected, and histopathological evaluation established the diagnosis of CHM. The patient was discharged in good condition, in which during his four-month follow-up period, no recurrence of the tumor has been observed.

**Conclusion:**

Although cavernous hemangioma rarely present in the mediastinum, it should be considered in the differential diagnosis of mediastinal tumors. However, our review of literature demonstrated a female dominance and average age of 40 years, with a 52% mortality rate based on previous reports.

## Background

Cavernous hemangioma (CH) is a common benign tumor with vascular origin. It can grow in many locations and its occurrence in some organs, like the liver, is so frequent that it is considered the most common primary tumor of the liver [1]. Nevertheless, an incidence rate of less than 0.5% makes cavernous hemangioma of mediastinum (CHM) a rare cause of mediastinal mass [2].

While there have been reports of CH in the middle mediastinum, the majority of hemangiomas tend to present in the anterior mediastinum [3, 4]. A preoperative definite diagnosis is somewhat challenging due to the rarity of CHM and its nonspecific clinical manifestation, and paraclinical findings and diagnosis are often made after total resection of the tumor, which is the mainstay of management [5, 6]. Here, we intend to present a case of CHM with no symptoms other than persistent cough.

## Case presentation

A 48-year-old man was referred to our center after dealing with a persistent severe cough for more than a month. Past medical history was positive for hypertension. He also had a recent episode of syncope which had been resolved spontaneously. Vital signs were within the normal range with an arterial oxygen saturation of 97%. Physical examinations were unremarkable including heart and lung examination. Laboratory findings were also unremarkable.

**Fig. 1:**
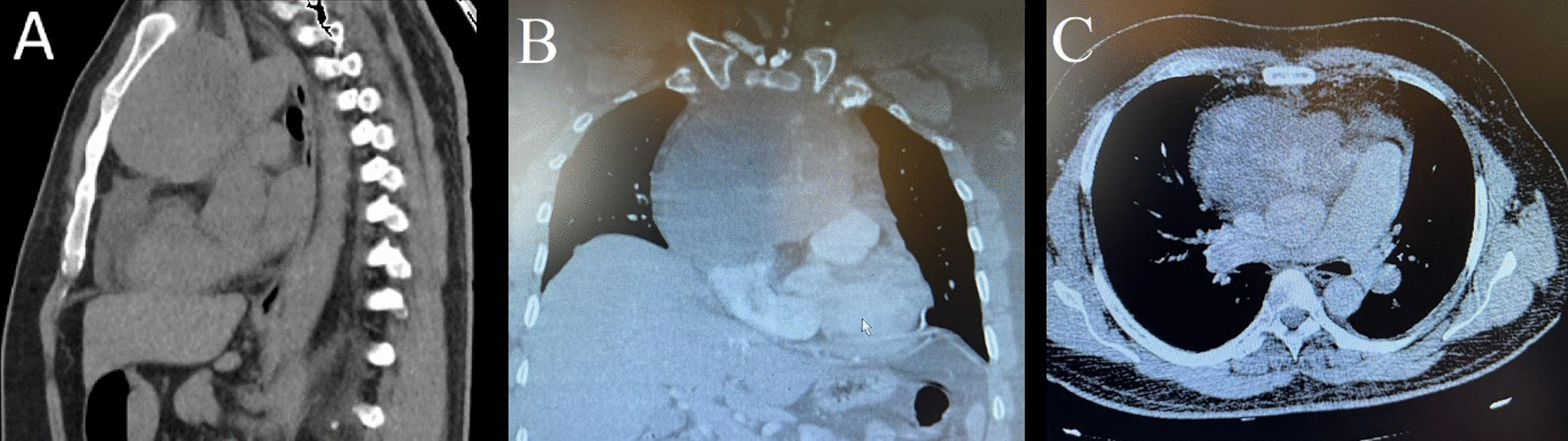
Chest computed tomography scan showing a large mediastinal mass; **A** Sagittal view, **B** coronal view, and **C** axial view

In order to evaluate his persistent cough, spiral chest computed tomography (CT) scan with intravenous contrast was performed which revealed a heterogeneous lobulated mass in the anterior superior mediastinum measuring 125 × 77 × 99 mm in size, with pressure over the aortic arch, and mild pericardial effusion (Fig. 1).

A tru-cut biopsy from the mass was reported as a benign vascular tumor. The patient was operated to resect the mass. During the operation a full sternotomy was conducted. The pleura was opened and a bilateral mediastinal pleurectomy was performed. The tumor was then dissected from the diaphragm, as well as pericardium. Due to encasement of innominate vein by the mass, a dissection of the tumor and innominate vein was performed, while preserving both phrenic nerves.

**Fig. 2:**
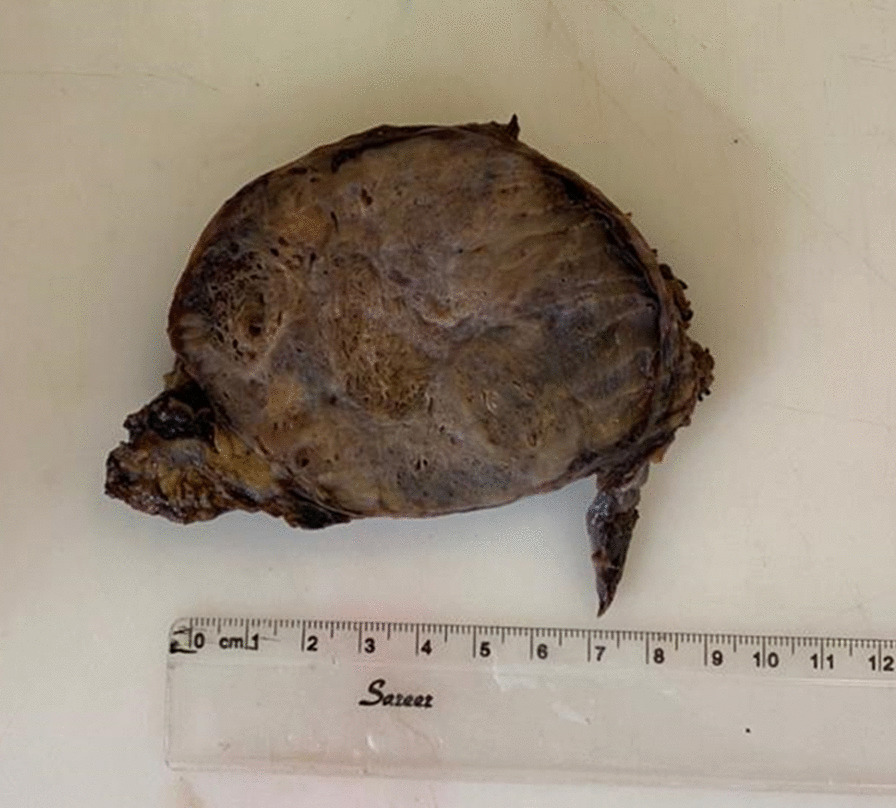
Gross lobulated mass in the anterior mediastinum identified as cavernous hemangioma

**Fig. 3:**
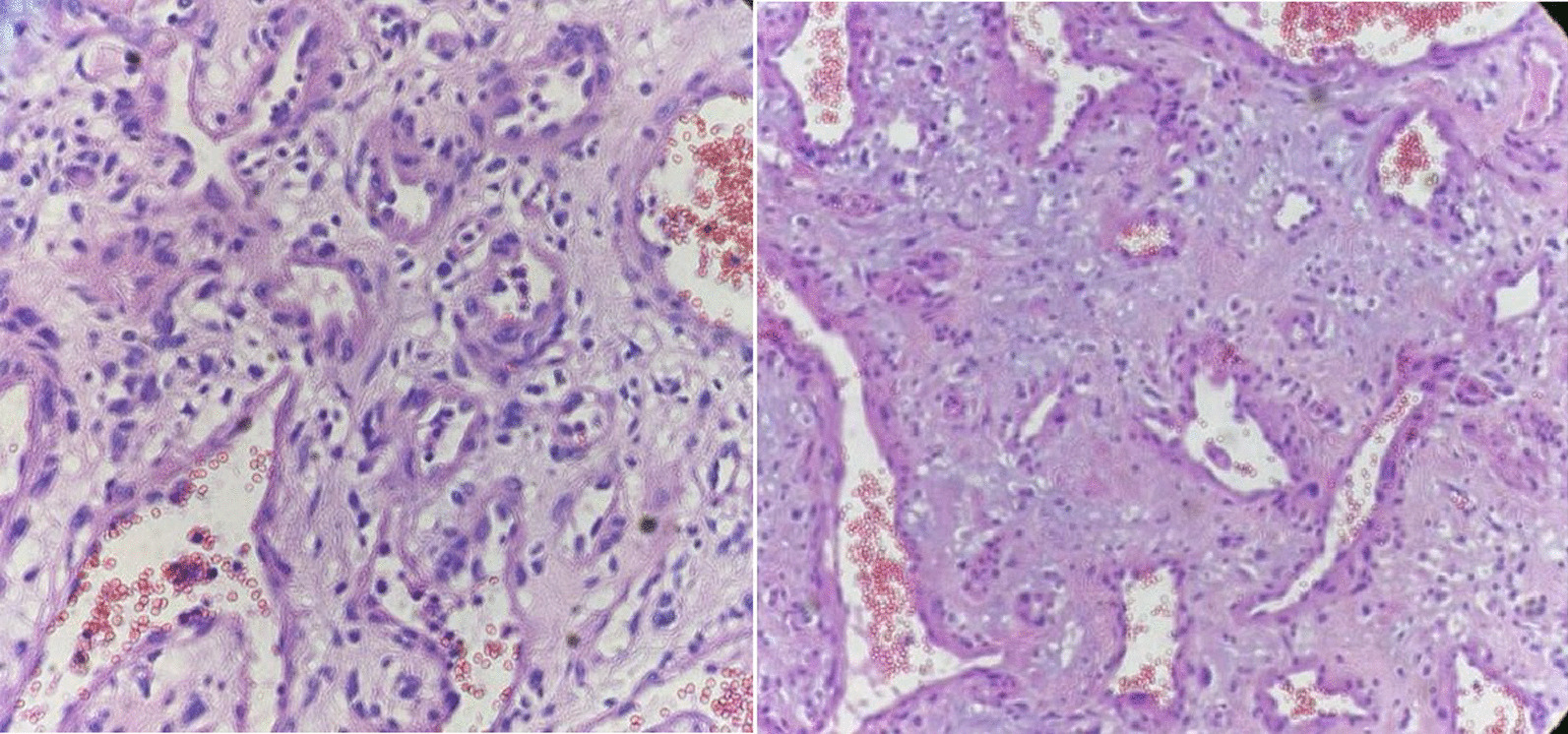
H&E staining of anterior mediastinum mass demonstrating large and dilated vascular channels filled with blood, with vascular spaces lined by normal and bland looking endothelial cells, typical for cavernous hemangioma

The specimen was sent for pathologic examination. The gross examination of the tissues obtained from surgery showed a bosselated brown soft tissue (12 × 11 × 6 cm) and a fragment of fibro-fatty tissue (7.5 × 4 × 3 cm) (Fig. 2). Cut section of the tumor was hyper-vascular filled with blood, exhibiting brown color. Microscopic histopathological evaluation of the tumor showed large and dilated vascular channels filled with blood. The vascular spaces were lined by normal and bland looking endothelial cells. These histopathologic features were typical for CH. A reactive lymph node was also identified (Fig. 3).

**Fig. 4:**
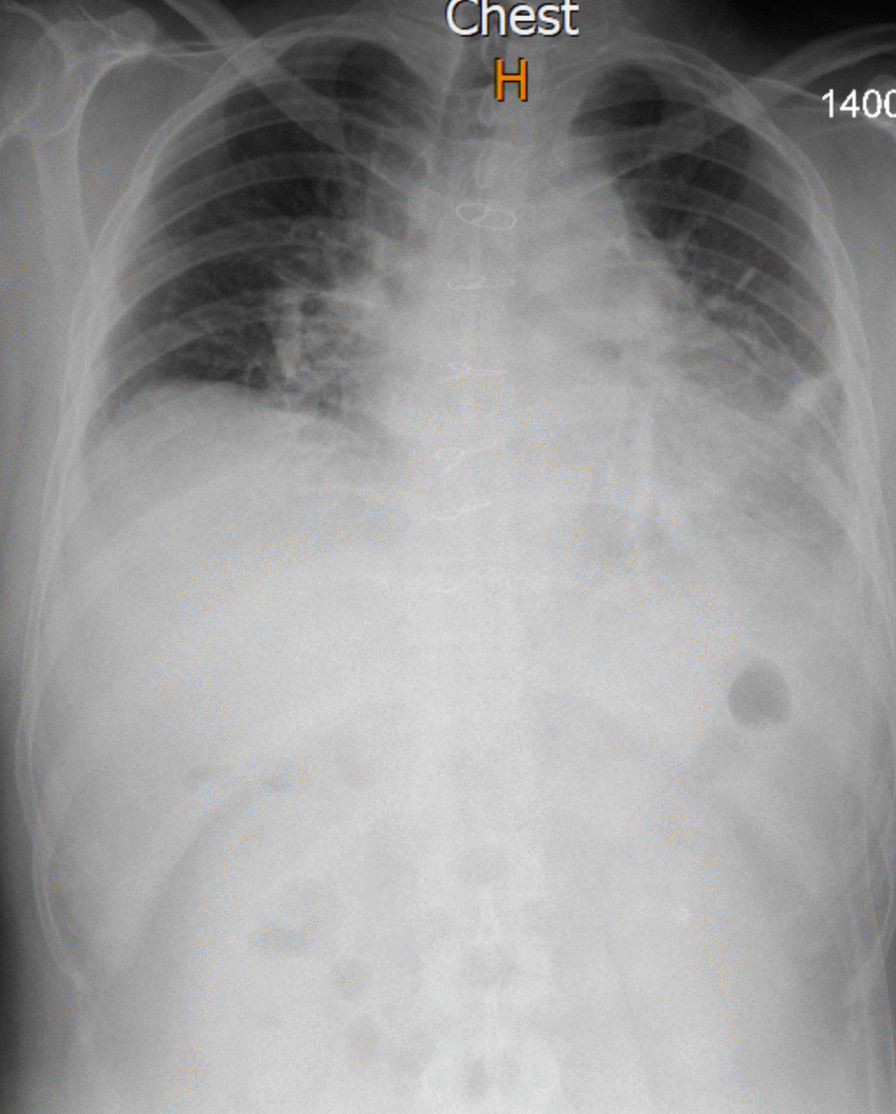
Post-operative chest X-ray of 48-year-old male patient undergoing Cavernous Hemangioma of mediastinum tumoral resection

The patient had an uncomplicated surgery and a postoperative chest X-ray was unremarkable (Fig. 4). The patient was discharged in good condition a week after the operation. The patient is relatively well and completely symptom free during his fourth month follow-up.

## Discussion

Cavernous Hemangioma of mediastinum (CHM) is a rare benign tumor originating from vascular endothelial cells. It is mainly an internally endothelium lined multilocular mass consisted from cavernous sinusoids of varying size [2, 7]. A rare cause of mediastinal mass is CHM, which usually involves the anterior mediastinum [2, 5]. However, there have been reports of posterior [8, 9], middle [3, 4] and even concurrent anterior and middle [10] mediastinum involvements. We further investigated all reports regarding CHM among the major databases of PubMed, Scopus, Web of Science, and Google Scholar, based on related Mesh terms (“Cavernous Hemangioma”, and “Mediastinal” or similar phrases) till before October 2022. The related reports are described in Table 1.

**Table 1:** Literature review of mediastinal tumor cases

References	Number of cases	Age (years), gender	Symptoms	Location of tumor	Imaging modalities and finding	Tumor size (mm)	Treatment	Outcome (follow-up)
Abuharb [11]	1	52, M	1-year of chest tightness and shortness of breath	Epicardium, just over the anterior wall of the right ventricular outflow tract	CTA: a mass on epicardium	30 × 20	Surgical resection	Alive (3 months)
Akiba [12]	1	27, M	Cough	Anterior mediastinum	X-ray: left mediastinal widening, CT: anterior mediastinal mass with some phleboliths and swelling of the left innominare vein, 3D CT: dilation of innominate vein	50 × 45 × 30	Surgical resection	NA
Ampollini [13]	1	71, F	Incidental radiological finding	Left costovertebral space in contact with ascending aorta	X-ray: left paravertebral pulmonary opacity, MRI: a well-defined, oval-shaped lesion, placed in the left costovertebral space in contact with the ascending aorta	50 × 30 × 25	Surgical resection	Alive (60 months)
Arizono [14]	1	36, F	3-weeks back pain	Along the thoracic and the abdominal esophagus, from the level of carina to the cardiac junction	CECT: a poorly enhanced or nonenhanced mediastinal soft tissue mass, with an irregular margin along the thoracic and the abdominal esophagus, from the level of the carina to the cardiac junction, MRI: multilocular lesion existed surrounding the esophagus, Endoscopy: a blue color lesion distributed throughout mucosa with normal surface pattern, andicationg blood-containing submucosal tissue	107 × 66 × 43	NA	NA
Ashino [15]	1	74, F	Anterior chest pain and palpitation	Mediastinum around heart	X-ray: mild enlargement of heart, Echocardiograph: tumor lay in the mediastinum	NA	Surgical resection	Alive (12 months)
Bagheri [16]	1	15, M	Chronic cough and neck swelling	Anterior mediastinum	CT: an anterior mediastinal mass with extension to the neck with a density of 0–30 HU along with calcification and phleboliths	NA	Surgical resection	NA
Cai [4]	1	57, M	Incidental finding	Middle mediastinom	CT: a circular low-density lesion in the mediastinum, MRI: irregular abnormal signals in the vena cava-anterior trachea space, with uniformly low signals on T1WI and significantly high signals on T2WI	60 × 50 × 30	Surgical resection	Alive (12 months)
Chen [17]	1	42, F	Exertional chest pain as well as lower extremity edema	Anterior mediastinum originating from the right atrioventricular groove and abutting the superior vena cava, right atrium, right ventricular outflow tract and aortic root	CT and MRI: a large mass in the anterior mediastinum originating from the right atrioventricular groove and abutting the superior vena cava, right atrium, right ventricular outflow tract and aortic root	92 × 57 × 35	Three doses of bevacizumab (which failed) then Surgical resection	NA
Das [18]	1	56, F	6-months right-sided dull aching chest pain	Right posterior mediastinom	X-ray: a right paracardiac opacity, CT: homogeneous mass lesion of soft tissue density in right posterior mediastinum	65 × 53	Surgical resection	NA
Das [19]	1	7, M	Incidental radiological finding	Middle mediastinom extending from carina to the diaphragm in the pathway of the azygous vein	Enhanced MDCT: a gradually enhancing giant middle mediastinal vascular mass extending from the carina to the diaphragm in the pathway of the azygous vein	80 × 70	nothing	NA
Dixon [20]	1	43, M	Incidental radiological finding	Left anterior mediastinum at the level of the arch of the aorta	X-ray: a dense mass situated in the anterior mediastinum, antero-posterior and lateral tomograms confirmed the presence of a spherical mass approximately 9 cm Bronchoscopy, bronchography, barium swallow and pulmonary angiography were normal	110 × 70 × 40	Surgical resection	Alive (8 months)
Dobritoiu [21]	1	52, F	Asymptomatic, incidentally detected during a routine abdominal ultrasound	Middle mediastinum in contact with the right atrium	TTE and TEE confirmed the presence of a giant mass located in the middle mediastinum, in contact with the right atrium and interatrial septum; The cardiac MRI revealed a solid mediastinal mass with well-defined margins; CT scan showed that the tumor was in close contact with all three lobes of the right lung, and with the right hemi- diaphragm in the cardiophrenic angle	120 × 80 × 76	Surgical resection	NA
Drevet [22]	1	69, F	Persistent dyspnea	Roof of the right atrium, In the pericardium but extra-cardiac	CT: homogeneous tissular mass, adjacent to the right atrium; Contrast‐enhanced MRI in morphokinetic sequences in the right ventricular outflow tract confirmed a homogeneous, hypervascular and encapsulated mass in the roof of the right atrium, in the pericardium but extra‐cardiac, associated with a laminated superior vena cava; 18‐FDG PET scan showed an isolated low and heterogeneous hypermetabolism of the lesion with a standardized uptake value of 1.8	80 × 70 × 40	Surgical resection	Alive (60 months)
Guo [23]	1	45, F	Chest distress and dyspnea for 7 years	Anterior mediastinal space	MRI: huge lesion at the anterior mediastinal space	215 × 84 × 98	Surgical resection	NA
Jhan [24]	1	44, F	Incidental radiological finding	Posterior mediastinum near the head of the left third and fourth ribs	CT: a mass located in the posterior mediastinum near the heads of the left third and fourth ribs	24 × 22 × 12	Surgical resection and Sclerotherapy with histoacryl tissue coagulant in lipidol	NA
Joobeur [25]	1	46, M	Chest pain after trauma (public road accident.)	Left anterior mediastinum	CT scan: a hypodense left anterior mediastinal tissue mass measuring cm in size	23 × 19	Surgical resection	NA
Kaya [26]	1	56, M	Back pain for the last three weeks	Left paracardiac mass	X-ray and CT: A left paracardiac mass of 18 cm in diameter	180	Surgical resection	NA
Kim [27]	1	61, M	Incidental radiological finding	Anterior and middle mediastinum	CT: thin-walled multiloculated cystic masses in the anterior mediastinum, and a mass with both cystic and soft tissue attenuation with punctate calcifications in the middle mediastinum	NA	Surgical resection of most of them	Alive (48 months)
Kuo [28]	1	30, M	Intermittent cough with scanty sputum for 1 year	Between the superior vena cava and azygous vein	CT: a mass with only partial enhancement located between the superior vena cava and azygous vein MRI: a lesion low in signal intensity on the T1 image and high in SI on the T2 image with intense enhancement after gadolinium diethylene-triamine penta-acetic acid injection Angiography: hypervascular tumor in the right paratracheal area which was supplied by the right bronchial artery	NA	Surgical resection	NA
Li [29]	1	60, F	Right neck mas showing gradual enlargment for half a year	Right superior mediastinum	X-ray: a bulging mass in right superior mediastinum causing left-sided tracheal deviation, CT scan: a low attenuation and circumscribed mass in right superior mediastinum CT scan with contrast: the mass revealed initially peripheral nodular enhancement with gradually central fill-in on the delayed phase images	NA	Surgical resection	NA
Li [30]	1	26, M	Incidental radiological finding	Right mediastinum close to the epicardium of the right atrium	CT: a mass in the right mediastinum	65 × 60 × 50	Surgical resection	Alive (13 months)
Li [31]	1	38, F	Three-week history of intermittent cough and dull aching chest pain	Right Common pulmonary artery, the intraoperative exploration revealed an anterior mediastinal pericardial mass	CT: a heterogeneous mass with soft-tissue density interspersed with a fatty ingredient in the right common pulmonary artery, with well-defined and circumscribed margin	52 × 46 × 45	Surgical resection	NA
Lim [32]	1	26, M	Sudden onset left side chest pain and mild dyspnea since the morning	Left side of anterior mediastinum	X-ray: a mass in the left side of the anterior mediastinum and left side pleural effusion CT: a well-circumscribed ovoid soft tissue mass in the left side of the anterior mediastinum and left side pleural effusion	85 × 60 × 50	surgical resection	Alive (5 months)
Liu [33]	1	50, F	Incidental radiological finding	Left anterior mediastinum	CT: a non-enhancing mass in the left anterior mediastinum	90 × 70 × 60	Surgical resection	Alive (3 months)
Lovrenski [34]	1	67, F	Incidental radiological finding due to pneumonia workups	Right upper lobe of lung	CT: an infiltrative mass in its greatest dimension in the right upper lobe	46	Surgical resection	NA
Nakada [35]	1	43, M	Chest pain	Anterior mediastinum	CT: an anterior mediastinal tumor with a focal speck of calcification, composed of low-density soft tissue mass along with a remarkably dilated left innominate vein	60 × 52 × 38	Surgical resection	Alive (5 months)
Nchimi [36]	1	70, F	Acute orthopnea and mild retrosternal pain	Anterior to the great vessels of heart and right ventricle	Echocardiography: a large solid hypoechoic mass anterior to the ascending aorta and root of the pulmonary artery, resulting in compression of the anterior aspect of the right ventricle CT: homogeneous and well-delineated mass, isodense to the heart, located anterior to the great vessels and the right ventricle After contrast: heterogeneous and asymmetrical enhancement of the mass, with a peripheral nodular pattern	70 × 70 × 50	Surgical resection	Alive (6 months)
Okochi [37]	1	50, F	Incidental finding	Left anterior mediastinum, adjacent to the aortic arch	CT: a well-demarcated, spherical, nodular lesion in her left anterior mediastinum, adjacent to the aortic arch Dynamic contrast- enhanced CT revealed peripheral nodular enhancement within the lesion in the arterial phase, showing the same degree of contrast as in the aortic arch	28 × 26 × 26	Surgical resection	NA
Ose [38]	1	71, F	Pericardial discomfort while coughing	Left lateral mediasrtinal mass	CT: a left lateral mediastinal mass	23 × 18 × 13	Surgical resection	NA
Ouladdameshghi [39]	1	36, F	Dyspnea in the last three months	Right anterior mediastinum	X-ray: mediastinal widening, indicating the presence of a mediastinal mass CT: a heterogeneous lesion in the right anterior mediastinum	100 × 100 × 60	Surgical resection	NA
Roldan-Banos [40]	1	59, F	Incidental radiological finding	Anterior mediastinum	CT: a mass in the anterior mediastinum	50 × 40	Surgical resection	Alive (33 months)
Rotaru [41]	1	9, F	Exertional dyspnea that has been gradually increasing during the last year	Antero-superior mediastinum	X-ray: an abnormal mediastinal shadow, the finding being suspicious of a right upper mediastinal mass CT: well-circumscribed ovoid mass in the antero-superior mediastinum	84 × 50 × 44	Surgical resection	NA
Shen [42]	1	52, M	Incidental radiological finding	Anterior mediastinum	CT: a circumscribed soft tissue mass that was located in the anterior mediastinum, contrast-enhanced CT: a demarcated soft tissue mass with no calcification and marginal enhancement	22 × 12 × 8	Surgical resection	Alive (8 months)
Yun [9]	1	58, F	Intermittent back pain	Left posterior mediastinum	X-ray: a smooth, round mass in the left upper lung field CT: a left posterior mass, which abutted the 5th thoracic vertebra and descending aorta	60 × 50	Surgical resection	Alive (18 months)
Zheng [43]	1	30, F	Incidental radiological finding	Anterior mediastinum	CT: an anterior mediastinal oval tumor with border regularity and without necrosis and calcification	23 × 17 × 13	Surgical resection	Alive (12 months)
Feinberg [8]	1	8, M	Incidental finding	Left mediastinum	Fluoroscopy: a non-pulsating left paravertebral mediastinal fusiform mass was evident, extending from the mid-dorsal level to and through the diaphragm	NA	Surgical resection	NA
Gindhart [44]	1	38, M	Incidental finding	Anterior–superior mediastinum above the arch of the aorta, to the right of the trachea and esophagus at the level of the second to fourth thoracic vertebrae	Tomograms revealed no calcification and indicated that the mass was solid without cystic components or communication with the vertebral column Thyroid scan was normal as were tests of thyroid function. Barium swallow was normal	40	Surgical resection	Alive (8 months)
Hanaoka [45]	1	5, M	Cough and high fever	Left lung field	X-ray: an abnormal shadow in the left lung field, CT: a lurg multilocular cystic tumor without calcification arising from the anterior mediastinum, MRI: a multilocular cystic tumor with a heterogeneous high signal intensity on both T1 and T2 weighted images	105 × 60 × 60	Surgical resection	Alive (126 months)
Igari [46]	1	4, F	Cough	Anterior mediastinum	Post mortal CT and MRI: a giant mass occupied the anterior mediastinum, compressing the heart toward the left dorsal side and deforming the tracheal rings	130 × 130 × 70	Autopsy finding	Mortality (Autopsy finding)
Ishii [47]	1	7, F	Incidental radiological finding	Left anterior mediastinum	X-ray: large mediastinal massive shadow that enlarged to the left side of the left hilum CT: a left-sided anterior mediastinal mass in contact with the aorta and the chest wall with soft tissue density area and low-density area	60 × 40 × 20	Surgical resection	NA
Kissin [48]	1	29, F	Cough	Upper mediastinum	X-ray: a superior mediastinal shadow on the right and many phleboliths in the soft tissue of the thoracic cage in the right axilla Thyroid scan was normal. An arch aortagram showed no arteriovenous shunt in the mass	NA	nothing	NA
Kotoulas [49]	1	62, F	Palpitation	Posterior atrial wall	Heart ultrasound: a myxoma-like left atrium lesion	26 × 26 × 14	Angiographic resection	Alive (15 months)
Landolphi [50]	1	50, F	Increased dyspnea, more frequent palpitation and episodes of near syncope	Right atrium	Two-dimentional echocardipgraphy: a 4 to 5 Cm cystic unilocular mass adjacent to the right atrium MRI: soft tissue density within the right atrium	50 × 40	Surgical resection	NA
Lee [51]	1	23, M	Incidental radiological finding	Right lower-paratracheal area	X-ray: lesion in the right-paratracheal area. CT: well defined and homogeneously enhancing mass, in the patient's right upper posterior mediastinum	40 × 30 × 15	Surgical resection	NA
Mineo [52]	3	34, F	Left chest pain, weakness and shortness of breath	Posterior mediastinum	X-ray: an opacification of the left hemithorax with mediastinal shift to the right, CT: a huge mass in the posterior mediastinum occupying the upper portion of the left hemithorax	100 × 80	Surgical resection	Alive (48 months)
		20, M	Incidental radiological finding	Superior mediastinum	CT: a lesion in the superior mediastinum	60 × 40	Surgical resection	Alive (66 months)
		17, F	Orthopnea and persistent cough, (8 m ago: hoarseness and odynophagia)	Superior mediastinum	X-ray: an enlargement of the superior mediastinum, CT: a mass extensively involving the mediastinum and mild compression of the trachea	NA	Medication (prednisolone), just reduced the size of the tumor	NA
Moran [53]	18	48, F	2 Months of dyspnea	Anterior mediastinum	NA	40	Surgical resection	Alive (24 months)
		48, F	Cough	Posterior mediastinum	NA	30	Surgical resection	Alive (3 months)
		New born, M	Incidental finding	Anterior mediastinum	NA	20	Surgical resection	Alive (24 months)
		31, M	Incidental finding	Anterior mediastinum	NA	140	Surgical resection	Alive (36 months)
		1Mo, M	Rectal bleeding and telangiectasis on face	Posterior mediastinum	NA	NA	Autopsy finding	Mortality (Autopsy finding)
		37, M	Incidental finding	Posterior mediastinum	NA	90	Surgical resection	Alive (48 months)
		4Mo, M	Heart murmur	Anterior mediastinum	NA	20	Surgical resection	Alive (48 months)
		35, F	Neck pain	Posterior mediastinum	NA	70	Surgical resection	NA
		16, M	Abdominal pain	Anterior mediastinum	NA	20	Surgical resection	Alive (1 months)
		2, M	Cough	Anterior mediastinum	NA	100	Surgical resection	Alive (12 months)
		41, F	Chest pain	Anterior mediastinum	NA	80	Surgical resection	Alive (12 months)
		74, M	Na	Anterior mediastinum	NA	30	Surgical resection	NA
		20, M	Chest pain	Anterior mediastinum	NA	20	Surgical resection	NA
		34, F	Chest pain	Anterior mediastinum	NA	40	Surgical resection	NA
		36, M	Na	Anterior mediastinum	NA	30	Surgical resection	NA
		5Mo, F	Cough	Anterior mediastinum	NA	200	Piecemeal surgical excision	NA
		19, M	Incidental finding	Anterior mediastinum	NA	140	Surgical resection	Alive (24 months)
		68, F	Cough, chest pain	Anterior mediastinum	NA	40	Surgical resection	Alive (6 months)
Nakamura [54]	1	51, M	Swelling of the anterior chest	Anterior mediastinum	CT: a tumor developing in the anterior mediastinum and invading the anterior chest wall	30 × 25	Surgical resection	NA
Rosenberg [55]	1	9Mo, F	Shortness of breath	Upper right side of chest	Roentgenographic examination: a large, solid mass in the upper right side of the chest was to fill the cupola and extend into the thoracic inlet	40 × 25 × 20	Surgical resection	Alive (24 months)
Seline [56]	2	73, F	Incidental radiological finding	Posterior mediastinum	CT: a posterior mediastinal mass of the soft tissue density without calcifications	NA	Surgical resection	NA
		39, F	Progressive gait difficulty and decreased sensation of the right thigh and midthorax	Right upper paravertebral region	X-ray: a 3 cm right upper paravertebral mass, CT: a paravertebral mass with an intraspinal component	NA	Surgical resection	NA
Shikada [3]	1	51, F	Incidental radiological finding	Adjacent to the aortic arch	X-ray: a mass superimposed on the aortic arch, CT: a tumor with regular margin and homogeneous density adjacent to the aortic arch MRI: a mass with signal intensity approximately equal to the muscle on T1-weighted images and signal intensity distinctly higher than muscle on T2-weighted images	38 × 22	Surgical resection	Alive (10 months)
Ueno [57]	1	51, M	Esophagitis and discomfort in the back of the throat and abnormal nodule in the right lower lung field	Right lower lung field	CT: a homogeneous nodular opacity without calcification and an unclear border between the lesion and the diaphragm MRI: the tumor seemed to be an extrapulmonary lesion	17 × 10	Surgical resection	Alive (3 months)
Li [58]	1	64, F	Dizziness	Right ventricle	Echo: a solid lesion in the right ventricle	35 × 32	Surgical resection and Symptomatic support treatment	Alive (12 months)
Thilak [59]	1	55, F	Chest pain and breathlessness	Right atrium	TEE: homogenous, non-pedunculated mass arising from right atrial septum CT: a large hypodense lesion in the right atrium arising from the atrial septum, and the mass was partially occluding the Inferior Vena Cava	113 × 77 × 71	Surgical resection	Alive (1 months)
Toscano [60]	1	66, F	Fever, generalized myalgia, non-specific malaise, non-selective anorexia and choluria lasting for several weeks	Mitral valve	TEE: a large mass at the level of the posterior leaflet of the mitral valve	28 × 19	Surgical resection and valve replacement and Antibiotic therapy	Alive (8 months)
Turner [61]	1	29, F	Incidental finding, 3 years later came with chest and neck pain, dysphagia and dyspnea	Middle mediastinom (2017), right middle mediastinum extending to the right superior mediastinum (2020)	CT and MRI (2017): a large, cystic lesion posterior to the superior vena cava and lateral to the trachea and esophagus, CTA (2020): a large non-enhancing, low-attenuation mass in the right middle mediastinum extending to the right superior mediastinum	100 × 80 × 74	Surgical resection	NA
Vu [62]	1	71, M	Dull abdominal pain in the epigastrium, sometimes- sharp pain, spreading to the left chest and back, for about 1 month	Right atrium	TTE: a small tumor adhered to the right ventricular wall which changing shape with the heart contraction CT: showed no mass in the right ventricular chamber MRI: a small tumor with regular margins and well-defined boundaries	17 × 14 × 13	Surgical resection	NA
Grimes [63]	1	31, F	Headaches associated with nausea and vomiting	Posterior mediastinum	Roentgenographic study: a well-circumscribed, oval, soft, tissue mass in the posterior mediastinum, medial and posterior to the apex of the right lung	80 × 60	Surgical resection	NA
Mardani et al. (2021), Our case	1	48, M	Chronic cough (1 month)	Anterior mediastinum	CT: heterogeneous lobulated mass in the anterior superior mediastinum with pressure over the aortic arch, and mild pericardial effusion	125 × 77 × 99	Surgical resection	Alive (4 months)

CECT, contrast enhanced computerized tomography; CT, computerized tomography; CTA, computerized tomography angiography; F, female; M, male; Mo, months; MDCT, multidetector computerized tomography; MRI, magnetic resonance imaging; NA, not available; TEE, transesophageal echocardiography; TTE, transthoracic echocardiography

Based on the mentioned cases in Table 1 and our literature review, a total of 77 patients with mediastinal mass have been reported to date, with the oldest being reported on 1953 [63]. Among them, 31 (40.3%) were male and 46 (59.7%) were female. The age of the patients varied from a new born to 74 years with an average of 39.18 ± 21.67. Among the patients, 19 (24.7%) had no significant past medical history. The symptoms of the patients included cardiac symptoms in 19 (25.3%) and pulmonary symptoms in 26 (34.7%) of the patients, while also incidental in 26 (33.8%) of the cases. The most common location of the tumour was in the anterior mediastinum in 41 (53.2%) of cases, followed by middle mediastinum in 16 (20.8%) of cases, superior mediastinum in 16 (20.8%) of the cases, and posterior mediastinum in 12 (15.6%) of the cases. The size of the tumor ranged from 17 to 215 (median 56, Q1–Q3: 30–90.50), while the weight ranged from 20 to 1075 (median: 170, median 170, Q1–Q3: 52.0–657.5) grams. Treatment included surgical resection in 71 (92.2%) of the patients, in which two also received medication prior to surgery. Also, two (2.6%) patients solemnly received conservative management. The median follow-up duration was 12 [Q1–Q3: 6–33.75] months and the mortality rate was 40 (51.9%). Furthermore, two (2.6%) of the cases were diagnosed during autopsy.

A paper by Cohen et al. published in 1987 was the first comprehensive case series regarding CHMs. They presented 15 cases of CHM with 8 of them being asymptomatic [2]. This is consistent with a recent retrospective systematic review conducted by Li et al., when 52 percent of their studied cases were asymptomatic, discovered during routine checkups and imaging studies performed for other reasons [6]. Aside from the asymptomatic cases, clinical presentation usually correlates with the location of the tumor in the mediastinum and the compression it puts on the adjacent structures [2]. Symptoms include cough, dyspnea, chest pain, hemoptysis, plural effusion, and in some extreme situations, CHMs can present as congestive heart failure [39, 64]. The persistent cough noted in our patient is unique due to the fact it was the sole presenting symptom. A similar case in a study by Bagheri et al. presented not only with cough, but also with a large neck mass [16].

CHM’s imaging finding usually indicates a round to oval-shaped mass with a well-defined border, but it can also invade adjacent organs and this renders the tumor border to be hazy to an extent that it can be confused with a malignant lesion [7, 65]. The presence of phleboliths, pampiniform growth pattern and aberrant draining veins are relatively specific in diagnosing CHMs [6]. Magnetic resonance imaging can be helpful in CT suspected cases of CHM [7]. Markedly high intensity on fat suppression T2-weighted image might be a characteristic feature of CHMs [66]. Fatty components in the peripheral region of tumor is a sensitive indicator for CHM, but a central fatty component is not sensitive as it can also be seen in teratomas [6]. Table 2 demonstrates most common causes of anterior mediastinal masses in a comparative style.

**Table 2:** Differential diagnosis of anterior mediastinal masses

Pathology	Radiological findings (computed tomography scan)	Tumor characteristics	History, sign and symptoms
Mediastinal goitre	Inhomogeneous density with cystic areas and calcifications Markedly contrast-enhancement [67]	Encapsulated and lobulated mass with inhomogeneous appearance with cystic areas and calcifications [67]	Asymptomatic Compressive symptoms (e.g., dyspnea) [67]
Thymoma	Soft-tissue attenuation Mild to moderate contrast enhancement [67]	Oval, round or lobulated mass Cystic or necrotic degeneration when large Capsular calcification [67]	Asymptomatic Myasthenia gravis related symptoms (i.e., rapid fatigue) Compressive symptoms (e.g., dyspnea) [67]
Lymphoma	Homogeneous soft-tissue mass Mild to moderate contrast enhancement Pleural and pericardial effusions [67]	Differs based on the subtype of lymphoma but generally they are large, smooth or lobulated, anterior mediastinal masses [68]	Constitutional symptoms (particularly in Hodgkin lymphoma) Compressive symptoms (e.g., dyspnea) [67]
Teratoma	Well-defined unilocular or multilocular cystic lesion containing fluid, soft tissue, and fat attenuation [67]	Well-differentiated benign tissues with predominant ectodermal element [67]	Usually asymptomatic [67]
Cavernous hemangioma of mediastinum	Phleboliths Pampiniform growth pattern Aberrant draining veins [6]	Internally endothelium lined multilocular mass consisted from cavernous sinusoids of varying size [2]	Asymptomatic Compressive symptoms (e.g., dyspnea) Chest pain Cough [39, 64]

Just like our case, a CHM diagnosis is usually established after surgery and examination of the excised tumor, and preoperative diagnosis is seldomly reached [9]. This was also evidenced by a case series of 18 patients by Xu et al. in which two cases had an accurate preoperative diagnosis. They concluded their study by describing their surgical approach to these patients. While eight of their cases were initially treated by video-assisted thoracic surgery, three of them required thoracotomy due to extensive hemorrhage. This made their total number of thoracotomies 13 out of 18 cases [69]. Hence, we used the more traditional approach of thoracotomy in our patient.

While it has been proposed that a subtotal resection would suffice without any increase in morbidity and mortality [2], we performed a total resection due to encasement of the innominate vein by tumor and adhesions to pleura.

There have been reports of additional treatments alongside surgery. Radiotherapy and extracorporeal membrane oxygenation has been used in cases of CHMs with low platelet counts (i.e. Kasabach–Merritt syndrome) and massive hemoptysis, respectively [70, 71].

## Conclusion

Cavernous Hemangioma, a rare cause of mediastinal mass, should be considered in the differential diagnosis of mediastinal tumors. Our review of literature demonstrated a female dominance and average age of 40 years, with a 52% mortality rate based on previous reports. While imaging study can be helpful in diagnosing these benign lesions, it often needs surgery for both definite diagnosis and treatment.

## Data Availability

All data regarding this case report has been reported in the manuscript. Please contact the corresponding author in case of requiring any further information.

## Abbreviations

CH: Cavernous hemangioma
CHM: Cavernous hemangioma of mediastinum
CT: Computed tomography
